# Deep learning reconstruction improves the image quality of low-dose temporal bone CT with otitis media and mastoiditis patients

**DOI:** 10.1016/j.heliyon.2023.e22810

**Published:** 2023-11-23

**Authors:** Tian-Jiao Wang, Yun Wang, Zhu-Hua Zhang, Ming Wang, Man Wang, Tong Su, Ying-Hao Xu, Zhuang-Fei Ma, Jian Wang, Yu Chen, Zheng-Yu Jin

**Affiliations:** a Department of Radiology, Peking Union Medical College Hospital, Chinese Academy of Medical Sciences, No. 1 Shuai Fu Yuan, Dong Cheng District, Beijing, 100730, China; b Canon Medical Systems (China) CO., LTD., Building 205, Yard NO. A10, JiuXianQiao North Road, Beijing, 100015, China

**Keywords:** Deep learning, Temporal bone, Radiation dosage, Multidetector computed tomography, Otitis media, Mastoiditis

## Abstract

**Objective:**

To evaluate the image quality of low-dose temporal bone computed tomography (CT) in otitis media and mastoiditis patients by using deep learning reconstruction (DLR).

**Materials and methods:**

A total of ninety-seven temporal bones from 53 consecutive adult patients who had suspected otitis media and mastoiditis and underwent temporal bone CT were prospectively enrolled. All patients underwent high resolution CT protocol (group A) and an additional low-dose protocol (group B). In group A, high resolution data were reconstructed by filter back projection (FBP). In group B, low-dose data were reconstructed by DLR mild (B1), DLR standard (B2) and DLR strong (B3). The objective image quality was analyzed by measuring the CT value and image noise on the transverse image and calculating the signal-to-noise ratio (SNR) on incudomallear joint, retroauricular muscle, vestibule and subcutaneous fat. Subjective image quality was analyzed by using a five-point scale to evaluate nine anatomical structures of middle and inner ear. The number of temporal bone lesions which involved in five structures of middle ear were assessed in group A, B1, B2 and B3 images.

**Results:**

There were no significant differences in the CT values of the four reconstruction methods at four structures (all p > 0.05). The DLR group B1, B2 and B3 had significantly less image noise and a significantly higher SNR than group A at four structures (all p < 0.001). The group B1 had comparable subjective image quality as group A in nine structures (all p > 0.05), however, the group B3 had lower subjective image quality than group A in modiolus, spiral osseous lamina and stapes (all p < 0.001), the group B2 had lower subjective image quality than group A in modiolus and spiral osseous lamina (both p < 0.05). The number of temporal bone lesions which involved in five structures for group A, B1 and B2 images were no significant difference (all p > 0.05), however, the number of temporal bone lesions which involved in mastoid for group B3 images were significantly more than group A (p < 0.05). The radiation dose of high resolution CT protocol and low-dose protocol were 0.55 mSv and 0.11 mSv, respectively.

**Conclusion:**

Compared with high resolution CT protocol, in the low-dose protocol of temporal bone CT, DLR mild and standard could improve the objective image quality, maintain good subjective image quality and satisfy clinical diagnosis of otitis media and mastoiditis patients.

## 1 Introduction

Computed tomography (CT) has become a major diagnostic technique for temporal bone imaging because its high spatial resolution is suitable for visualizing the small anatomic structures of the middle and inner ear [[1], [2], [3], [4]]. Temporal bone CT is a radiologic examination for inflammatory diseases such as otitis media and mastoiditis [5,6]. Some patients of otitis media and mastoiditis will become cholesteatomatous otitis media and mastoiditis. If the patients are diagnosed as cholesteatomatous otitis media and mastoiditis on CT images, surgery is required as soon as possible. Therefore, for patients of otitis media and mastoiditis, follow-up CT is needed for evaluation and judgement of the surgery opportunity. In diagnosis and follow-up of otitis media and mastoiditis, radiation exposure is focused by radiologists. If the radiation dose can be reduced as much as possible, the clinical application of temporal bone CT will be expanded. There have been many methods for reducing the radiation dose in CT, including low tube voltage, low tube current and automatic tube current modulation [[7], [8], [9]]. However, image quality and diagnostic accuracy are affected under low-dose conditions. Filter back projection (FBP) reconstruction is a conventional reconstruction method used in the clinical diagnosis of CT images. Recently, deep learning reconstruction (Advanced Intelligent Clear-IQ Engine [AiCE], Canon Medical Systems) uses deep convolutional neural networks (DCNNs) to exact signal from low-dose hybrid iterative reconstruction (HIR) images to have similar image quality as the high-dose model-based iterative reconstruction (MBIR) target [10]. So, even under low-dose condition, the deep learning reconstruction (DLR) images have the features of high-dose MBIR images. Some studies have reported that the image quality of DLR improved image quality in low-dose CT, for example cardiac and abdominal CT angiography [11,12]. However, to our knowledge, there was no previous study has reported the image quality of temporal bone CT with DLR.

Therefore, the purpose of this study was to evaluate the image quality of low-dose temporal bone CT in otitis media and mastoiditis patients by using DLR, compared with high resolution CT with FBP.

## 2 Materials and methods

This study was performed in patients enrolled at our institution, and this study was approved by the ethics committee of our hospital (HS-2812). All participants in this study provided written informed consent.

### 2.1 Patients enrollment

Consecutive adult patients who had suspected otitis media and mastoiditis without surgery, and underwent temporal bone CT from November 2020 to April 2021 were prospectively enrolled. The exclusion criteria were the following: (1) motion artifacts; (2) congenital deformity.

### 2.2 CT acquisition protocols

All temporal bone CT examinations were performed on a 320-row detector CT scanner (Aquilion ONE Genesis, Canon Medical System Corporation) using a volume scan with acquisition in a single volume covering the temporal bone region. To evaluate the image quality and diagnostic performance of otitis media and mastoiditis patients using DLR in low-dose temporal bone CT, all patients underwent temporal bone CT with the high resolution CT protocol and an additional low-dose protocol.

In high resolution CT protocol, the following parameters were used: 120 kV; 250 mAs; 1-s rotation time; 0.5 mm slice thickness; field of view (FOV), 110 mm; and image matrix, 512 × 512. The automatic exposure control was off, and the volume CT dose index was 32.5 mGy. Images were reconstructed by using the FBP algorithm with kernel FC81 (group A). Axial images were reconstructed with a 0.5 mm section thickness in 0.3 mm increments. Coronal and sagittal images were reconstructed with a 1 mm section thickness in 1 mm increments.

In low-dose protocol, the following parameters were used: 120 kV; 50 mAs; 0.5-s rotation time; 0.5 mm slice thickness; FOV, 110 mm; and image matrix, 512 × 512. The automatic exposure control was off, and the volume CT dose index was 6.7 mGy. Images were reconstructed by using DLR mild (group B1), DLR standard (group B2) and DLR strong (group B3). Axial images were reconstructed with a 0.5 mm section thickness in 0.3 mm increments. Coronal and sagittal images were reconstructed with a 1 mm section thickness in 1 mm increments.

### 2.3 Objective image analysis

**Fig. 1:**
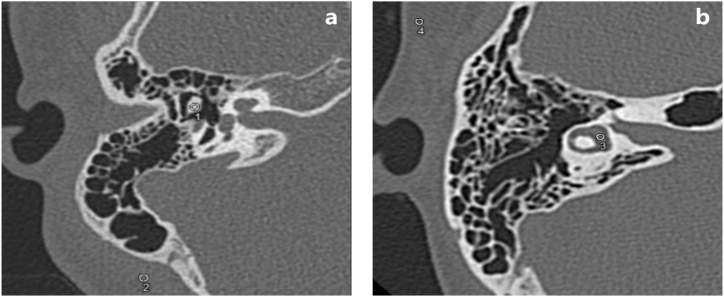
Examples of ROIs putting in incudomallear joint (1), retroauricular muscle (2), vestibule (3) and subcutaneous fat (4).

The objective image quality was evaluated by a radiologist with 5 years of experience in temporal bone CT diagnosis. The computed tomography attenuation value and image noise (standard deviation of attenuation value) were measured by placing circular regions of interest (ROIs) on axial images in the following four structures: incudomalleolar joint, retroauricular muscle, vestibule and subcutaneous fat, which were focused clinically (Fig. 1). We drew ROIs in similar areas in each location. The signal-to-noise ratio (SNR) was calculated by dividing the mean attenuation value by the image noise of each ROI.

### 2.4 Subjective image analysis

**Fig. 2:**
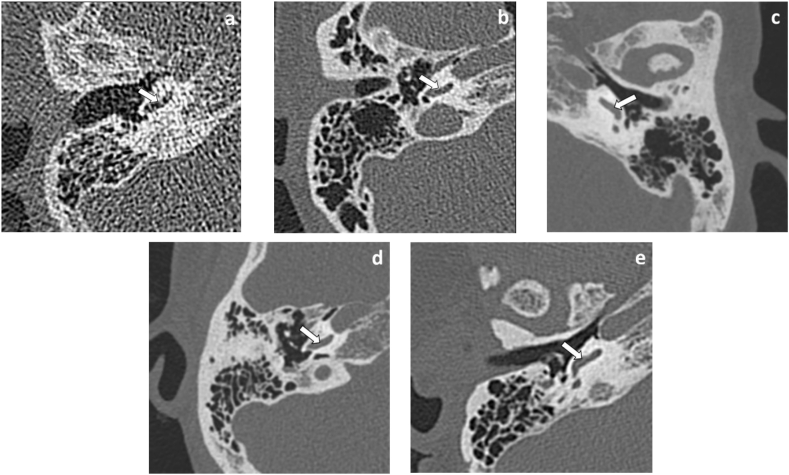
Examples of image score of 1, 2, 3, 4 and 5 in spiral osseous lamina (white arrow) of inner ear were shown in a, b, c, d and e, respectively.

The subjective image quality was independently evaluated by two radiologists (with 5 and 10 years of experience in temporal bone CT). Two radiologists were blinded to the scan parameters and reconstruction technique. Images were presented in a random order. In total, nine middle and inner ear anatomical structures (Table 1) were assessed by five-point scores: 1 = anatomical structures not identifiable due to poor image quality, 2 = structures identifiable but no assessable details, 3 = anatomical structures still fully assessable in all parts, 4 = clear delineation of structure, and 5 = very good delineation of structure [13]. Fig. 2 a, b c, d and e show examples of image scores of 1, 2, 3, 4 and 5, respectively. The radiologists could freely change the window level and window width and freely browse axial, coronal and sagittal images.

**Table 1:** Nine anatomical structures included in subjective image analysis.

Structure	Review Criteria
**Middle ear**	
Malleus	Presence of all parts
Incus	Presence of all parts
Stapes	Presence of all parts
Facial canal wall, tympanic segment	Contour, course
Mastoid	Bony borders, aeration
**Inner ear**	
Cochlea	Normal contour
Spiral osseous lamina	Presence, intensity
Modiolus	Presence, intensity
Facial canal wall, cochlear segment	Contour, course

### 2.5 Evaluation of temporal bone lesions

Evaluation of temporal bone lesions in middle ear and mastoid was performed by other two radiologists with 10 and 20 years of work experience in temporal bone CT diagnosis recording the number of inflammation which involved in malleus, incus, stapes, facial canal wall and mastoid for group A, B1, B2 and B3.

### 2.6 Radiation dose evaluation

The CT dose index (CTDIvol) and dose length product (DLP) for each patient were obtained from the patient dose report. The DLP was calculated by the product of CTDIvol and the scan range (cm). The effective dose (ED) was calculated for each scan by multiplying the DLP by the International Commission on Radiological Projection (ICRP) conversion factor of 0.0021 [mSv/(mGy ∙ cm)] for the head region of adults [14]. The CTDIvol, DLP and ED were recorded for the high resolution CT and low-dose protocols.

### 2.7 Statistical analysis

All statistical analysis were performed with SPSS (version 20.0; SPSS, Chicago, Ill) software. Categorical data are expressed as frequency and percentage; normal continuous data are expressed as the mean ± standard deviation; non-normal continuous data are expressed as media (Q1, Q3). The *t*-test and Mann-Whitney *U* test were performed to compare the differences in the objective and subjective image quality between reconstructed images of high resolution CT and low-dose protocols. Mann-Whitney *U* test was performed to compare the differences in evaluation of temporal bone lesions between reconstructed images of high resolution CT and low-dose protocols. Inter-observer and intra-observer agreement were calculated using the kappa statistic. The degree of agreement based on kappa values was as follows: 0.00–0.20, 0.21–0.40, 0.41–0.60, 0.61–0.80, or 0.81–1.00, indicating slight, fair, moderate, substantial, or almost perfect agreement, respectively [15]. All tests were two tailed, and a p value < 0.05 was considered indicative of statistical significance.

## 3 Results

**Fig. 3:**
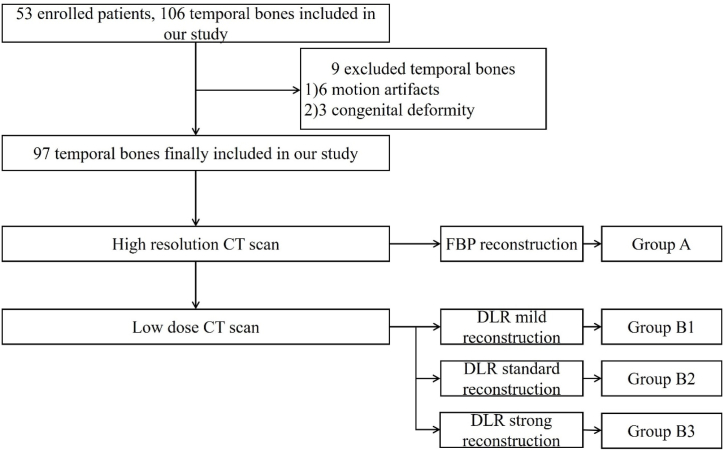
Flow chart of study design.

There were 24 males and 29 females, aged 18–83 years, with an average of 50 ± 8 years among 53 otitis media and mastoiditis patients. Totally, 106 temporal bones were included in this study. Nine temporal bones were excluded: three temporal bones with congenital deformity and six temporal bones with motion artifact. Finally 97 temporal bones were included in this study (Fig. 3). Until now, 34 otitis media and mastoiditis patients underwent follow-up CT evaluating the change of lesions. Results of follow-up CT showed 9 patients are complete response and 25 temporal bones are partial response.

### 3.1 Objective image analysis

**Fig. 4:**
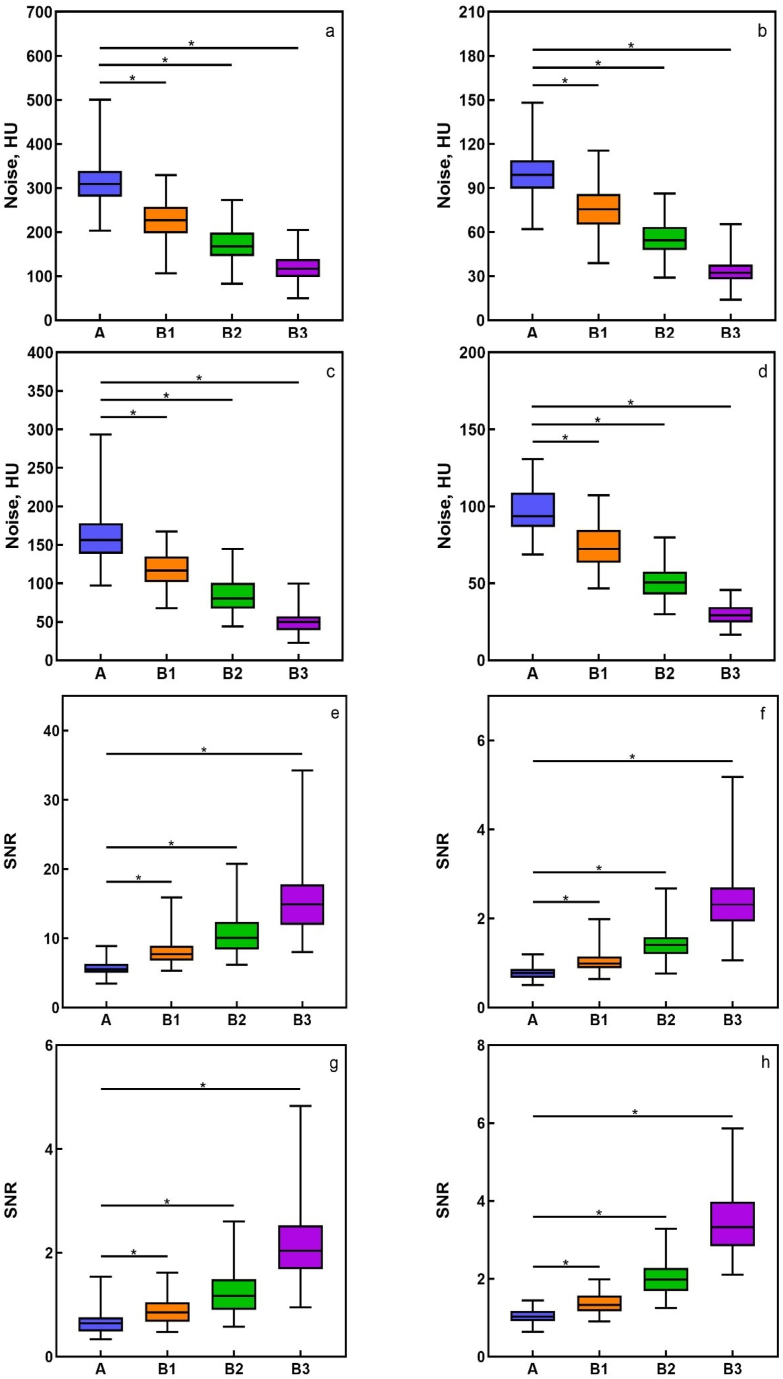
Image quality assessments according to image noise and SNR. Box and whisker plots a, b, c and d show incudomalleolar joint noise, retroauricular muscle noise, vestibule noise, and subcutaneous fat noise at group A, B1, B2 and B3, respectively. Box and whisker plots e, f, g and h show incudomalleolar joint SNR, retroauricular muscle SNR, vestibule SNR, and subcutaneous fat SNR at group A, B1, B2 and B3, respectively. The central line represents the median, box boundaries represent the first and third quartiles, and whisker boundaries extend 1.5 quartiles. * = p < 0.001 compared with group A.

The CT value of the four structures were non-significance using the four reconstruction methods (all p > 0.05), Group B1, B2 and B3 had significantly less image noise and a significantly higher SNR than group A at four structures (all p < 0.001) (Table 2). Image noise of group B3 was lowest and SNR of group B3 was highest (Fig. 4).

**Table 2:** The CT value, image noise and SNR of four structures using four reconstruction methods in 97 temporal bones.

	A	B1	B2	B3
**CT value(HU)**				
Incudomalleolar joint	1723.44 ± 113.90	1724.76 ± 114.26	1724.43 ± 113.05	1721.73 ± 114.09
Retroauricular muscle	75.95 ± 5.09	75.76 ± 5.28	75.75 ± 5.06	75.36 ± 4.70
Vestibule	98.99 ± 20.49	97.83 ± 19.80	98.27 ± 20.48	98.68 ± 19.92
Subcutaneous fat	−98.21 ± 5.89	−98.42 ± 6.23	−98.31 ± 6.18	−97.76 ± 6.05
**Image noise(HU)**				
Incudomalleolar joint	309.76 ± 50.24	224.36 ± 41.95*	171.99 ± 40.47*	119.10 ± 31.97*
Retroauricular muscle	100.49 ± 17.12	76.60 ± 14.73*	55.61 ± 11.46*	33.96 ± 9.01*
Vestibule	157.72 ± 32.82	117.23 ± 24.11*	84.98 ± 21.64*	49.74 ± 14.32*
Subcutaneous fat	97.51 ± 15.55	74.11 ± 14.19*	51.28 ± 10.99*	29.91 ± 6.81*
**SNR**				
Incudomalleolar joint	5.71 ± 1.00	8.01 ± 1.87*	10.65 ± 2.93*	15.76 ± 5.34*
Retroauricular muscle	0.78 ± 0.14	1.03 ± 0.22*	1.42 ± 0.34*	2.37 ± 0.64*
Vestibule	0.65 ± 0.19	0.87 ± 0.25*	1.23 ± 0.41*	2.15 ± 0.76*
Subcutaneous fat	1.03 ± 0.18	1.38 ± 0.28*	2.00 ± 0.43*	3.45 ± 0.84*

* = p < 0.001 compared with group A.

### 3.2 Subjective image analysis

**Fig. 5:**
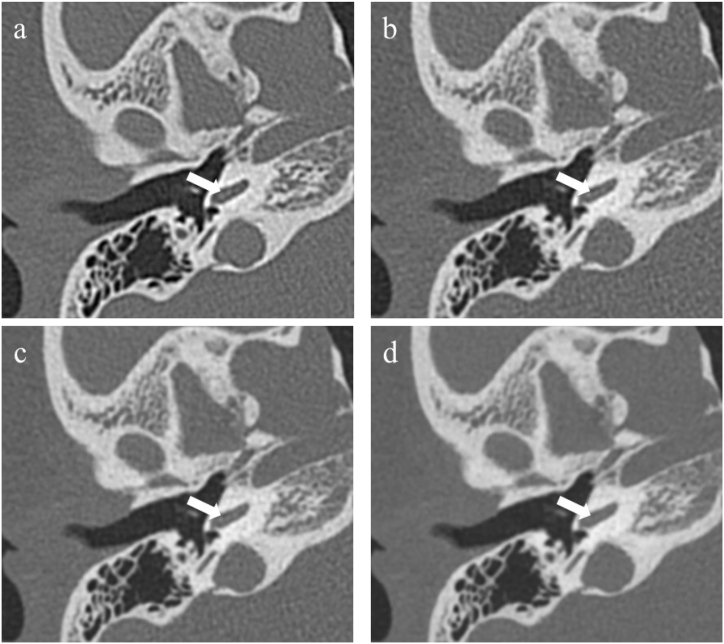
A 41-year-old female with suspected otitis media underwent temporal bone CT. The axial CT image scores of spiral osseous lamina in group A (a), B1 (b), B2 (c) and B3 (d) were 5, 5, 4 and 3, respectively. The conspicuity of the spiral osseous lamina was consistently and clearly shown on the group A and B1.

Two radiologists had almost perfect inter-observer and intra-observer agreement for the evaluation of subjective image quality (kappa = 0.973 and 0.936). The group B1 had same subjective image quality as group A in nine structures (all p > 0.05), however, the group B3 had lower subjective image quality than group A in modiolus, spiral osseous lamina and stapes (all p < 0.001), the group B2 had lower subjective image quality than group A in modiolus and spiral osseous lamina (both p < 0.05) (Table 3). Fig. 5 shows an example of conspicuity of the spiral osseous lamina in group A, B1, B2 and B3.

**Table 3:** Subjective image quality assessment in nine anatomical structures.

		A	B1	B2	B3	A vs B1	A vs B2	A vs B3
Malleus	5	89 (91.8 %)	88 (90.7 %)	88 (90.7 %)	86 (88.7 %)	0.800	0.800	0.470
	4	8 (8.2 %)	9 (9.3 %)	9 (9.3 %)	11 (11.3 %)			
	3	0 (0.0)	0 (0.0)	0 (0.0)	0 (0.0)			
	2	0 (0.0)	0 (0.0)	0 (0.0)	0 (0.0)			
	1	0 (0.0)	0 (0.0)	0 (0.0)	0 (0.0)			
Incus	5	90 (92.8 %)	88 (90.7 %)	87 (89.7 %)	87 (89.7 %)	0.603	0.447	0.447
	4	7 (7.2 %)	9 (9.3 %)	10 (10.3 %)	10 (10.3 %)			
	3	0 (0.0)	0 (0.0)	0 (0.0)	0 (0.0)			
	2	0 (0.0)	0 (0.0)	0 (0.0)	0 (0.0)			
	1	0 (0.0)	0 (0.0)	0 (0.0)	0 (0.0)			
Stapes	5	84 (86.6 %)	83 (85.6 %)	82 (84.5 %)	16 (16.5 %)	0.317	0.157	0.000
	4	13 (13.4 %)	14 (14.4 %)	15 (15.5 %)	64 (66.0 %)			
	3	0 (0.0)	0 (0.0)	0 (0.0)	17 (17.5 %)			
	2	0 (0.0)	0 (0.0)	0 (0.0)	0 (0.0)			
	1	0 (0.0)	0 (0.0)	0 (0.0)	0 (0.0)			
Facial canal wall, tympanic segment	5	93 (95.9 %)	91 (93.8 %)	91 (93.8 %)	90 (92.8 %)	0.517	0.517	0.353
	4	4 (4.1 %)	6 (6.2 %)	6 (6.2 %)	7 (7.2 %)			
	3	0 (0.0)	0 (0.0)	0 (0.0)	0 (0.0)			
	2	0 (0.0)	0 (0.0)	0 (0.0)	0 (0.0)			
	1	0 (0.0)	0 (0.0)	0 (0.0)	0 (0.0)			
Mastoid	5	94 (96.9 %)	94 (96.9 %)	94 (96.9 %)	94 (96.9 %)	1.000	1.000	1.000
	4	3 (3.1 %)	3 (3.1 %)	3 (3.1 %)	3 (3.1 %)			
	3	0 (0.0)	0 (0.0)	0 (0.0)	0 (0.0)			
	2	0 (0.0)	0 (0.0)	0 (0.0)	0 (0.0)			
	1	0 (0.0)	0 (0.0)	0 (0.0)	0 (0.0)			
Cochlea	5	87 (89.7 %)	85 (87.6 %)	85 (87.6 %)	85 (87.6 %)	0.651	0.651	0.651
	4	10 (10.3 %)	12 (12.4 %)	12 (12.4 %)	12 (12.4 %)			
	3	0 (0.0)	0 (0.0)	0 (0.0)	0 (0.0)			
	2	0 (0.0)	0 (0.0)	0 (0.0)	0 (0.0)			
	1	0 (0.0)	0 (0.0)	0 (0.0)	0 (0.0)			
Spiral osseous lamina	5	87 (89.7 %)	85 (87.6 %)	82 (84.5 %)	0 (0.0)	0.157	0.025	0.000
	4	10 (10.3 %)	12 (12.4 %)	15 (15.5 %)	39 (40.2 %)			
	3	0 (0.0)	0 (0.0)	0 (0)	52 (53.6 %)			
	2	0 (0.0)	0 (0.0)	0 (0.0)	6 (6.2 %)			
	1	0 (0.0)	0 (0.0)	0 (0.0)	0 (0.0)			
Modiolus	5	89 (91.8 %)	86 (88.7 %)	85 (87.6 %)	5 (5.2 %)	0.083	0.046	0.000
	4	8 (8.2 %)	11 (11.3 %)	12 (12.4 %)	71 (73.2 %)			
	3	0 (0.0)	0 (0.0)	0 (0.0)	21 (21.6 %)			
	2	0 (0.0)	0 (0.0)	0 (0.0)	0 (0.0)			
	1	0 (0.0)	0 (0.0)	0 (0.0)	0 (0.0)			
Facial canal wall, cochlear segment	5	93 (95.9 %)	91 (93.8 %)	91 (93.8 %)	91 (93.8 %)	0.517	0.517	0.517
	4	4 (4.1 %)	6 (6.2 %)	6 (6.2 %)	6 (6.2 %)			
	3	0 (0.0)	0 (0.0)	0 (0.0)	0 (0.0)			
	2	0 (0.0)	0 (0.0)	0 (0.0)	0 (0.0)			
	1	0 (0.0)	0 (0.0)	0 (0.0)	0 (0.0)

### 3.3 Evaluation of temporal bone lesions

**Fig. 6:**
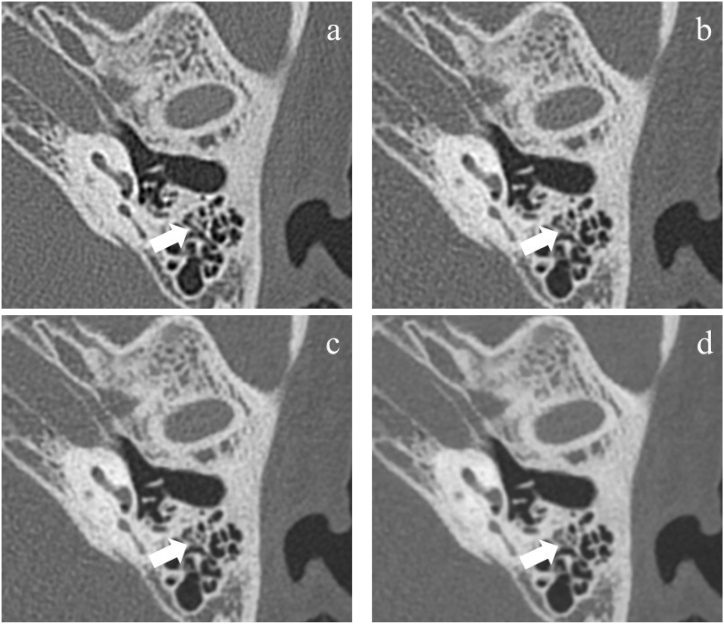
A 50-year-old male with suspected mastoiditis underwent temporal bone CT. Group A (a), B1 (b) and B2 (c) show no inflammation involved in mastoid. Group B3 (d) show a little inflammation in mastoid. Group B1 and B2 show good consistency with group A.

Two radiologists had good inter-observer and intra-observer agreement for the evaluation of temporal bone lesions (kappa = 0.882 and 0.841). Compared with group A, B1 and B2, group B3 significantly increased the number of lesions in mastoid (p < 0.001) (Table 4). Fig. 6 shows an example of assessment of mastoid lesion in group A, B1, B2 and B3.

**Table 4:** Number of temporal bone lesions which involved in malleus, incus, stapes, facial canal wall and mastoid with different reconstruction techniques.

	A	B1	B2	B3	A vs B1	A vs B2	A vs B3
Malleus	31 (31.9 %)	32 (32.9 %)	32 (32.9 %)	33 (34.0 %)	0.878	0.878	0.761
Incus	27 (27.8 %)	28 (28.8 %)	28 (28.8 %)	28 (28.8 %)	0.874	0.874	0.874
Stapes	25 (25.7 %)	26 (26.8 %)	27 (27.8 %)	29 (29.9 %)	0.871	0.746	0.523
Facial canal wall, tympanic segment	33 (34.0 %)	34 (35.0 %)	34 (35.0 %)	34 (35.0 %)	0.880	0.880	0.880
Mastoid	49 (50.5 %)	51 (52.5 %)	52 (53.6 %)	70 (72.1 %)	0.774	0.667	0.002

Data are presented as n (%).

### 3.4 Radiation dose

The CTDIvol, DLP and ED of the high resolution CT protocol were 32.5 mGy, 260.3 mGy ∙ cm and 0.55 mSv, respectively. The CTDIvol, DLP and ED of the low-dose protocol were 6.7 mGy, 53.3 mGy ∙ cm and 0.11 mSv, respectively. Therefore, the radiation dose of the low-dose protocol was reduced by 80 % compared with that of the high resolution CT protocol.

## 4 Discussion

Temporal bone lesions, such as otitis media and mastoiditis, lead to osseous inflammation and purulence. Clinically, CT imaging is the essential imaging modality for screening and diagnosing lesions of the temporal bone. As CT examination exposes patients to radiation, techniques that allow a dose reduction without decreasing the image quality and diagnostic performance would be useful in temporal bone CT, especially for otitis media and mastoiditis patients of follow-up check. In this study, DLR could decrease image noise and improve the image quality of low-dose temporal bone CT in otitis media and mastoiditis patients.

Akihiro Tada reported that low-dose temporal bone CT (effective dose: 0.21 mSv) provided the same diagnostic quality for the assessment of middle and inner ear anatomy, similar to that provided by the standard dose protocol [16]. In this study, consistent results were obtained but the radiation dose of low-dose protocol (effective dose: 0.11 mSv) was smaller than that in previous study. Kim and Jeon published research comparing image quality by using a low-dose protocol (150 kV Sn) with a standard dose protocol (120 kV) [13]. In the low-dose protocol, the effective dose was 0.26 mSv. However, in this study, the effective dose in the low-dose protocol was 0.11 mSv, which is smaller than that in previous study.

The results of this study showed that objectively, group B1, B2 and B3 had non-significance CT value as group A and that group B3 had lowest image noise and highest SNR. Subjectively, the image scores of the group B3 were lower than those of group A in the spiral osseous lamina, modiolus and stapes. The difference in group B1, B2 and B3 was different reconstruction intensity of DLR. Group B3 had the highest reconstruction intensity which would increase training process and reconstruction time. Subjectively, images of group B3 had the “plastic-like” appearance because of the higher reconstruction intensity have more similar image quality as the MBIR images which could not reduce the low-frequency image noise components [[17], [18], [19]]. Kurokawa also illustrated the MBIR images have lower image quality than HIR images in spiral osseous lamina [20]. Clinically, “plastic-like” image would decrease subjective acceptance especially in tiny structures [21]. Therefore, group B1 had higher subjective image score than group B3 in showing spiral osseous lamina, modiolus and stapes.

This study evaluated the number of temporal bone lesions which involved five middle ear structures with different reconstruction techniques. In assessment of otitis media, group A, B1, B2 and B3 had good consistency. In evaluation of mastoiditis, group A, B1 and B2 had good consistency, group B3 significantly increased the number of mastoiditis. High reconstruction intensity of DLR would decrease image noise and increase subjective smoothness of image, so tiny bone structures were waxy and over smooth and the some normal bone trabecula were mistaken for lesions. Standard reconstruction intensity of DLR did not affect the diagnosis performance of otitis media and mastoiditis. According to previous studies, low-dose temporal bone CT could be applied in assessment of normal middle and inner ear structures in infants, children and adults [13,16,22]. According to the results of this study, low-dose temporal bone CT with DLR could perform preoperative diagnosis which included evaluating variety and range of lesions in otitis media and mastoiditis patients. Besides, low-dose temporal bone CT with DLR could observe change of lesions in follow-up CT.

Our study has some limitations. First, only 53 patients were included in this study, and this study was a single-center study. Second, validation data were not collected in this study. Third, pathological results were not included because of no surgery in these patients. Fourth, high resolution CT is one of standard for diagnosis of otitis media and mastoiditis in our hospital, however, high resolution CT is not gold standard in clinical.

In conclusion, compared with high resolution CT, DLR (mild, standard and strong) could improve the objective image quality, DLR (mild and standard) maintain the subjective image quality and have good consistency for evaluation of lesions in low-dose temporal bone CT with otitis media and mastoiditis patients.

## Data availability statement

This research data cannot be shared at this time because the data also forms part of an ongoing study.

## Additional information

No additional information is available for this paper.

## Funding statement

Dr. Yu Chen was supported by 10.13039/501100001809National Natural Science Foundation of China (82001814).

Dr. Zhuhua Zhang was supported by 10.13039/5011000147642021 SKY Imaging Research Fund of the Chinese International Medical Foundation (Z-2014-07-2101).

## CRediT authorship contribution statement

**Tian-Jiao Wang:** Writing – original draft, Methodology. **Yun Wang:** Formal analysis, Conceptualization. **Zhu-Hua Zhang:** Visualization, Investigation, Funding acquisition. **Ming Wang:** Data curation. **Man Wang:** Data curation. **Tong Su:** Visualization, Validation. **Ying-Hao Xu:** Software, Resources. **Zhuang-Fei Ma:** Software, Resources. **Jian Wang:** Software, Resources. **Yu Chen:** Writing – review & editing, Funding acquisition. **Zheng-Yu Jin:** Supervision, Project administration.

## Declaration of competing interest

The authors declare the following financial interests/personal relationships which may be considered as potential competing interests: Ying-Hao Xu, Zhuang-Fei Ma and Jian Wang are employees of 10.13039/100015650Canon Medical Systems, China. The remaining authors declare no relationships with any companies whose products or services may be related to the subject matter of the article.
